# Tris(5,6-dimethyl-1,10-phenanthroline-κ^2^ *N*,*N*′)copper(II) bis­(hexa­fluorido­phosphate) acetonitrile monosolvate

**DOI:** 10.1107/S1600536812028267

**Published:** 2012-06-30

**Authors:** Yanis Toledano-Magaña, Juan-Carlos García-Ramos, Consuelo García-Manrique, Marcos Flores-Alamo, Lena Ruiz-Azuara

**Affiliations:** a Instituto de Investigaciones Biomédicas, Universidad Nacional Autónoma de México, Coyoacán 04510, DF, México; b Facultad de Química, Universidad Nacional Autónoma de México, Coyoacán 04510, DF, México

## Abstract

In the title compound, [Cu(C_14_H_12_N_2_)_3_](PF_6_)_2_·CH_3_CN, the [Cu(5,6-dmp)_3_]^2+^ cationic complex (5,6-dmp is 5,6-dimethyl-1,10-phenanthroline) is stabilized by two hexa­fluorido­phosphate anions and one acetonitrile solvent mol­ecule. The coordination geometry around the Cu^II^ atom can be described as distorted elongated octa­hedral with *R*_out_ = 2.277 (2) Å, *R*_in_ = 2.052 (2) Å and a tetra­gonality of 0.9011, acquiring a ‘static’ stereochemistry. In the supra­molecular network, there are inter­molecular C—H⋯F and C—H⋯N inter­actions with *R*_3_^3^(16), *R*_2_^2^(7), *R*_1_^2^(4), *R*_3_^3^(16) and *C*_3_^2^(7) motifs that lead to an infinite three-dimensional network.

## Related literature

For literature on metal complexes with phenanthroline-based ligands related to their intense luminescence, their capacity to inter­act with DNA and also in some cases the induction of DNA cleavage, see: Bencini & Lippolis (2010 ▶). For details of octa­hedral distortion and motifs, see: Ramakrishnan & Palaniandavar (2008 ▶); Murphy *et al.* (2006 ▶); Etter *et al.* (1990 ▶).
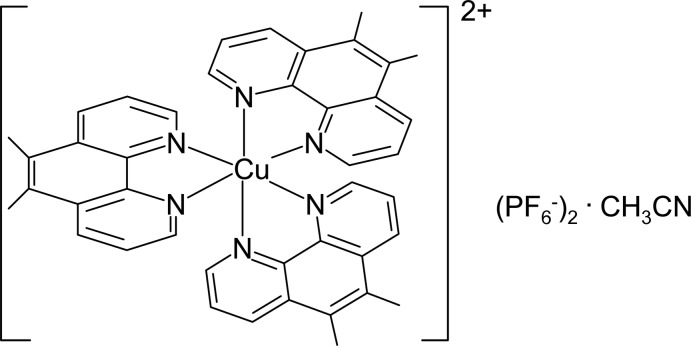


## Experimental

### 

#### Crystal data

[Cu(C_14_H_12_N_2_)_3_](PF_6_)_2_·C_2_H_3_N
*M* *_r_* = 1019.3
Monoclinic, 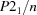

*a* = 9.8566 (3) Å
*b* = 19.9317 (7) Å
*c* = 22.1822 (6) Å
β = 93.603 (3)°
*V* = 4349.3 (2) Å^3^
*Z* = 4
Mo *K*α radiation
μ = 0.67 mm^−1^
*T* = 130 K
0.34 × 0.21 × 0.09 mm

#### Data collection

Oxford Diffraction Xcalibur, Atlas, Gemini diffractometer
Absorption correction: analytical (*CrysAlis PRO*; Oxford Diffraction, 2009 ▶) *T*_min_ = 0.643, *T*_max_ = 0.84
20467 measured reflections
8596 independent reflections
5782 reflections with *I* > 2σ(*I*)
*R*_int_ = 0.037

#### Refinement

*R*[*F* ^2^ > 2σ(*F* ^2^)] = 0.039
*wR*(*F* ^2^) = 0.097
*S* = 0.92
8596 reflections
602 parameters
H-atom parameters constrained
Δρ_max_ = 0.99 e Å^−3^
Δρ_min_ = −0.42 e Å^−3^

### 

Data collection: *CrysAlis CCD* (Oxford Diffraction, 2009 ▶); cell refinement: *CrysAlis RED* (Oxford Diffraction, 2009 ▶); data reduction: *CrysAlis RED*; program(s) used to solve structure: *SHELXS97* (Sheldrick, 2008 ▶); program(s) used to refine structure: *SHELXL97* (Sheldrick, 2008 ▶); molecular graphics: *ORTEP-3 for Windows* (Farrugia, 1997 ▶); software used to prepare material for publication: *WinGX* (Farrugia, 1999 ▶).

## Supplementary Material

Crystal structure: contains datablock(s) global, I. DOI: 10.1107/S1600536812028267/ru2037sup1.cif

Structure factors: contains datablock(s) I. DOI: 10.1107/S1600536812028267/ru2037Isup2.hkl

Additional supplementary materials:  crystallographic information; 3D view; checkCIF report

## Figures and Tables

**Table:** 

Cu1—N1	2.0063 (19)
Cu1—N1*A*	2.0144 (19)
Cu1—N2*A*	2.091 (2)
Cu1—N2*B*	2.095 (2)
Cu1—N2	2.220 (2)
Cu1—N1*B*	2.333 (2)

**Table:** 

N1*A*—Cu1—N2*A*	80.60 (8)
N1—Cu1—N2	78.35 (8)
N2*B*—Cu1—N1*B*	75.18 (8)

**Table 2:** Hydrogen-bond geometry (Å, °)

*D*—H⋯*A*	*D*—H	H⋯*A*	*D*⋯*A*	*D*—H⋯*A*
C1—H1⋯F1	0.95	2.3	3.149 (3)	148
C1*A*—H1*A*⋯N3^i^	0.95	2.74	3.489 (5)	136
C9—H9⋯F11^i^	0.95	2.62	3.270 (3)	126
C10—H10⋯F7^i^	0.95	2.54	3.358 (3)	145
C10—H10⋯F8^i^	0.95	2.59	3.471 (3)	155
C59—H59*C*⋯F4^ii^	0.98	2.3	3.265 (4)	170
C8*B*—H8*B*⋯F1^iii^	0.95	2.51	3.437 (3)	164
C3—H3⋯F8^iv^	0.95	2.47	3.392 (3)	163
C3*A*—H3*A*⋯F4^v^	0.95	2.62	3.557 (3)	169

Symmetry codes: (i) 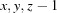; (ii) 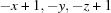; (iii) 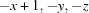; (iv) 
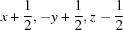
; (v) 
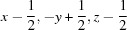
.

**Fig. 1.**
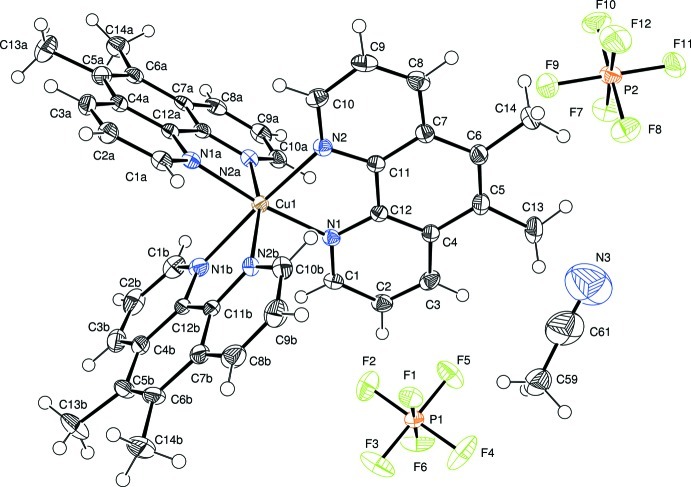
The molecular structure and the atom labelling scheme for (1). Displacement ellipsoids are draw at the 50% probability level and H atoms are shown as circles of arbitrary size.

**Fig. 2.**
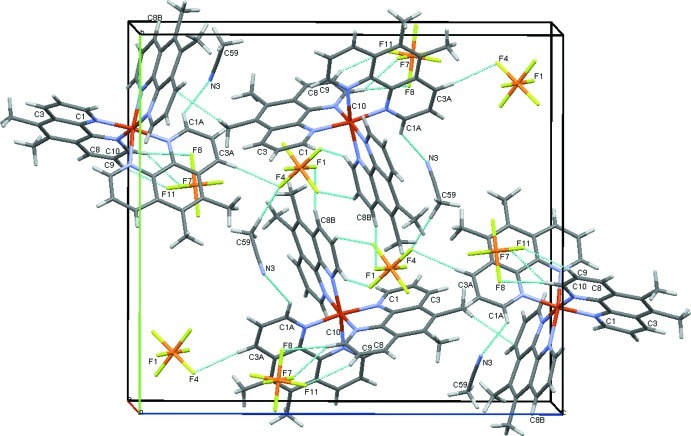
Intermolecular contacts of type hydrogen bond (dashed lines) in the crystal of (1,) forming infinite ribbons of R33(16), R22(7), R12(4), R33(16) and C32 (7) motif's.

## supplementary crystallographic information

### Comment

Due the combination of structural and chemical properties, metal complexes with phen-based ligands have been actively studied for their catalytic, redox, photochemical and photophysical properties and, more recently, as building units for the construction of efficient luminescent materials and even photoswitchable molecular devices (Bencini & Lippolis, 2010). Here, we present the crystal structure of the title compound rac-[Cu(5,6-dmp)_3_](PF_6_)_2_ CH_3_CN 1.

The X-ray structure of 1 consist of both Λ- and Δ-enantiomers of copper(II) complex cation, the molecular structure with crystallographic atom numbering scheme is illustrated in Fig. 1. Selected bond distances and bond angles are given in Table 1. The coordination geometry around Cu(II) can be described as distorted elongated octahedral (DEO) with N1, N1A, N2A, N2B nitrogen atoms occupying the corners of the square plane and N1B and N2 atoms occupying the *trans* axial positions. The distances (Cu1–N1B, 2.333 (2) Å; Cu1–N2, 2.220 (2) Å) mean Cu–N_out_ =*R*_out_ = 2.2766 (22) Å, are longer than the mean of the four in-plane Cu–N bond distances with Cu1–N1, 2.0063 (19); Cu1–N1A, 2.0144 (19); Cu1–N2A, 2.091 (2); Cu1–N2B, 2.095 (2) Å and mean of Cu–N_in_ = *R*_in_ = 2.0516 (20) Å. The average Cu–N bond distance (2.1641 (21) Å) is significantly longer than that [2.137 (4) Å] observed (Ramakrishnan & Palaniandavar, 2008) for *rac*-[Cu(5,6-dmp)_3_](ClO_4_)_2_ and very similar than that (2.189 (13) Å) observed (Murphy *et al.*, 2006) for the *rac*-[Cu(phen)_3_](ClO_4_)_2_. Interestingly, the tetragonality (T =*R*_in_/*R*_out_ = 0.9011) of 1 is shorter than that (0.952) of its *rac*-[Cu(5,6-dmp)_3_](ClO_4_)_2_ analogue suggesting that the former complex 1 acquires a *static* stereochemistry. Also, the bite angles of 5,6-dmp ligands in 1 (80.60 (8), 78.35 (8), 75.18 (8)°) deviate significantly from the ideal angle of 90°, which is consistent with the distorted coordination geometry. The average value (78.04°) of bite angles is less than that (78.5 °) (Murphy *et al.*, 2006) for the *rac*-[Cu(phen)_3_]^2+^ analogue, which is in completely agreement with the stronger coordination of the 5,6-dmp ligand.

The hexafluorophosphate ion and acetonitrile solvent are not involved in the coordination sphere of the Cu ion, but are in the crystal lattice. In the supramolecular network there are C—H···F and C—H···N intermolecular interactions of type hydrogen bond (Table 2) that help stabilize crystal packing (Fig. 2). The intermolecular interactions C1A—H1A..N3, C59—H59..F4 and C8B—H8B··· F1 are forming the *R*_3_^3^(16) motif. In addition, the hydrogen bond type formed from the donor-aceptor atoms: C3—H3···F8, C9—H9..F11, C10—H10···F7, C9A—H9A···F7 and C10—H10..F8 are forming the *R*_2_^2^(7), *R*_1_^2^(4), *R*_3_^3^(16) and *C*_3_^2^ (7) motif's mainly (Etter *et al.*, 1990). All these interactions lead to infinite three-dimensional network superstructure.

### Experimental

1 mmol (232 mg) of hemi-pentahydrated Cu(NO_3_)_2_ was dissolved in 5 ml of MeOH and mixed with 10 ml of ethanol solution of 5,6-dimethyl-1,10-phenanthroline (3 mmol, 625 mg). After 2 h of strong stirring, the resulting emerald green solution was mixed with three equivalents of ammonium hexafluorophosphate (3 mmol, 489 mg) resulting in a green powder, washed several times with cold water to eliminate the NH_4_PF_6_ excess. Once dry, the green product was isolated with 89% yield (870 mg). The product was redissolved in EtOH and the solvent was slowly evaporated to get suitable single crystals. Anal. calcd. for C_42_H_36_N_6_P_2_F_12_Cu (978.24 g/mol): C, 51.56; H, 3.70; N, 8.59. Found: C, 51.02; H, 3.81; N, 8.67. IR (KBr disc, cm^-1^):3412 br, 3067 br, 2923 m, 1621 m, 1605 m, 1583 s, 1523 m, 1481 m, 1430 m, 1358 s,, 875 s, 728 m.

### Refinement

H atoms attached to C atoms were placed in geometrically idealized positions, and refined as riding on their parent atoms, with C—H distances fixed to 0.95 (aromatic CH) and 0.98 (methyl CH_3_) and with *U*_iso_ of 1.2 and 1.5 *U*_eq_(C) respectively.

### Crystal data

**Table:**

[Cu(C_14_H_12_N_2_)_3_](PF_6_)_2_·C_2_H_3_N	*F*(000) = 2076
*M**_r_* = 1019.3	*D*_x_ = 1.557 Mg m^−^^3^
Monoclinic, *P*2_1_/*n*	Mo *K*α radiation, λ = 0.71073 Å
*a* = 9.8566 (3) Å	Cell parameters from 6486 reflections
*b* = 19.9317 (7) Å	θ = 3.4–26.0°
*c* = 22.1822 (6) Å	µ = 0.67 mm^−^^1^
β = 93.603 (3)°	*T* = 130 K
*V* = 4349.3 (2) Å^3^	Block, blue
*Z* = 4	0.34 × 0.21 × 0.09 mm

### Data collection

**Table:**

Oxford Diffraction Xcalibur, Atlas, Gemini diffractometer	8596 independent reflections
Graphite monochromator	5782 reflections with *I* > 2σ(*I*)
Detector resolution: 10.4685 pixels mm^-1^	*R*_int_ = 0.037
ω scans	θ_max_ = 26.1°, θ_min_ = 3.4°
Absorption correction: analytical (*CrysAlis PRO*; Oxford Diffraction, 2009)	*h* = −11→12
*T*_min_ = 0.643, *T*_max_ = 0.84	*k* = −24→19
20467 measured reflections	*l* = −27→22

### Refinement

**Table:**

Refinement on *F*^2^	Primary atom site location: structure-invariant direct methods
Least-squares matrix: full	Secondary atom site location: difference Fourier map
*R*[*F*^2^ > 2σ(*F*^2^)] = 0.039	Hydrogen site location: inferred from neighbouring sites
*wR*(*F*^2^) = 0.097	H-atom parameters constrained
*S* = 0.92	*w* = 1/[σ^2^(*F*_o_^2^) + (0.0518*P*)^2^] where *P* = (*F*_o_^2^ + 2*F*_c_^2^)/3
8596 reflections	(Δ/σ)_max_ = 0.001
602 parameters	Δρ_max_ = 0.99 e Å^−^^3^
0 restraints	Δρ_min_ = −0.42 e Å^−^^3^

### Special details

**Table:**

Geometry. All s.u.'s (except the s.u. in the dihedral angle between two l.s. planes) are estimated using the full covariance matrix. The cell s.u.'s are taken into account individually in the estimation of s.u.'s in distances, angles and torsion angles; correlations between s.u.'s in cell parameters are only used when they are defined by crystal symmetry. An approximate (isotropic) treatment of cell s.u.'s is used for estimating s.u.'s involving l.s. planes.
Refinement. Refinement of *F*^2^ against ALL reflections. The weighted *R*-factor *wR* and goodness of fit *S* are based on *F*^2^, conventional *R*-factors *R* are based on *F*, with *F* set to zero for negative *F*^2^. The threshold expression of *F*^2^ > 2σ(*F*^2^) is used only for calculating *R*-factors(gt) *etc*. and is not relevant to the choice of reflections for refinement. *R*-factors based on *F*^2^ are statistically about twice as large as those based on *F*, and *R*- factors based on ALL data will be even larger.

### Fractional atomic coordinates and isotropic or equivalent isotropic displacement parameters (Å^2^)

**Table:**

	*x*	*y*	*z*	*U*_iso_*/*U*_eq_	
C1	0.5532 (3)	0.21466 (13)	0.11579 (11)	0.0210 (6)	
H1	0.6285	0.2048	0.0926	0.025*	
C1A	0.3305 (3)	0.27265 (14)	−0.13015 (10)	0.0219 (6)	
H1A	0.2777	0.234	−0.1227	0.026*	
C1B	0.7496 (3)	0.24812 (15)	−0.03627 (11)	0.0297 (7)	
H1B	0.7672	0.2916	−0.0201	0.036*	
C2	0.5593 (3)	0.19719 (14)	0.17668 (10)	0.0245 (6)	
H2	0.6372	0.175	0.1945	0.029*	
C2A	0.3324 (3)	0.29670 (14)	−0.18916 (10)	0.0245 (6)	
H2A	0.2821	0.2744	−0.2212	0.029*	
C2B	0.8519 (3)	0.21597 (17)	−0.06489 (12)	0.0355 (8)	
H2B	0.9374	0.2372	−0.0681	0.043*	
C3	0.4527 (3)	0.21216 (14)	0.21058 (11)	0.0263 (6)	
H3	0.4563	0.2002	0.2521	0.032*	
C3A	0.4067 (3)	0.35210 (14)	−0.20058 (11)	0.0243 (6)	
H3A	0.4094	0.3683	−0.2408	0.029*	
C3B	0.8290 (3)	0.15336 (16)	−0.08846 (11)	0.0316 (7)	
H3B	0.8985	0.1309	−0.1083	0.038*	
C4	0.3372 (3)	0.24522 (12)	0.18435 (10)	0.0188 (6)	
C4A	0.4801 (3)	0.38584 (13)	−0.15286 (10)	0.0201 (6)	
C4B	0.7020 (3)	0.12232 (14)	−0.08313 (10)	0.0250 (6)	
C5	0.2224 (3)	0.26558 (14)	0.21789 (11)	0.0244 (6)	
C5A	0.5594 (3)	0.44604 (14)	−0.16013 (11)	0.0249 (6)	
C5B	0.6704 (3)	0.05518 (15)	−0.10550 (11)	0.0310 (7)	
C6	0.1182 (3)	0.30107 (13)	0.19016 (11)	0.0241 (6)	
C6A	0.6233 (3)	0.47694 (13)	−0.11138 (11)	0.0223 (6)	
C6B	0.5512 (3)	0.02559 (14)	−0.09430 (11)	0.0295 (7)	
C7	0.1167 (3)	0.31453 (13)	0.12576 (10)	0.0203 (6)	
C7A	0.6171 (3)	0.44820 (13)	−0.05164 (11)	0.0201 (6)	
C7B	0.4483 (3)	0.06267 (13)	−0.06392 (10)	0.0239 (6)	
C8	0.0110 (3)	0.34946 (13)	0.09339 (12)	0.0258 (6)	
H8	−0.0647	0.3654	0.1137	0.031*	
C8A	0.6846 (3)	0.47501 (14)	0.00109 (11)	0.0247 (6)	
H8A	0.7379	0.5145	−0.0014	0.03*	
C8B	0.3204 (3)	0.03597 (15)	−0.05259 (11)	0.0329 (7)	
H8B	0.299	−0.009	−0.0636	0.039*	
C9	0.0173 (3)	0.36040 (14)	0.03297 (12)	0.0269 (6)	
H9	−0.0529	0.3846	0.0112	0.032*	
C9A	0.6732 (3)	0.44432 (14)	0.05564 (11)	0.0263 (6)	
H9A	0.719	0.4622	0.091	0.032*	
C9B	0.2267 (3)	0.07456 (16)	−0.02572 (12)	0.0341 (7)	
H9B	0.1411	0.0562	−0.0171	0.041*	
C10	0.1275 (3)	0.33591 (13)	0.00339 (11)	0.0229 (6)	
H10	0.1298	0.3431	−0.0389	0.028*	
C10A	0.5942 (3)	0.38676 (14)	0.05904 (11)	0.0238 (6)	
H10A	0.5863	0.3662	0.0973	0.029*	
C10B	0.2580 (3)	0.14107 (14)	−0.01109 (11)	0.0279 (7)	
H10B	0.1914	0.168	0.0064	0.033*	
C11	0.2236 (3)	0.29286 (12)	0.09263 (10)	0.0172 (5)	
C11A	0.5413 (3)	0.38987 (13)	−0.04406 (10)	0.0182 (6)	
C11B	0.4733 (3)	0.12973 (13)	−0.04594 (10)	0.0203 (6)	
C12	0.3380 (3)	0.25989 (12)	0.12250 (10)	0.0164 (5)	
C12A	0.4711 (2)	0.35829 (12)	−0.09492 (10)	0.0171 (5)	
C12B	0.6041 (3)	0.15872 (13)	−0.05341 (10)	0.0194 (6)	
C13	0.2285 (3)	0.24542 (17)	0.28391 (11)	0.0393 (8)	
H13A	0.1479	0.2624	0.3026	0.059*	
H13B	0.3104	0.2644	0.3047	0.059*	
H13C	0.2313	0.1964	0.2871	0.059*	
C13A	0.5722 (3)	0.47206 (16)	−0.22359 (11)	0.0389 (8)	
H13D	0.6152	0.5164	−0.2218	0.058*	
H13E	0.4816	0.4756	−0.2443	0.058*	
H13F	0.628	0.4411	−0.2458	0.058*	
C13B	0.7767 (4)	0.02161 (19)	−0.14158 (15)	0.0549 (10)	
H13G	0.8516	0.0057	−0.1141	0.082*	
H13H	0.8114	0.0539	−0.1701	0.082*	
H13I	0.7358	−0.0166	−0.1638	0.082*	
C14	0.0007 (3)	0.32836 (16)	0.22315 (12)	0.0369 (7)	
H14A	0.0139	0.3174	0.2662	0.055*	
H14B	−0.0842	0.3082	0.2065	0.055*	
H14C	−0.0037	0.3772	0.2182	0.055*	
C14A	0.7017 (3)	0.54104 (14)	−0.11791 (13)	0.0321 (7)	
H14D	0.7988	0.5308	−0.1188	0.048*	
H14E	0.6869	0.5706	−0.0836	0.048*	
H14F	0.6705	0.5634	−0.1556	0.048*	
C14B	0.5192 (4)	−0.04628 (15)	−0.11287 (13)	0.0424 (8)	
H14G	0.4664	−0.0465	−0.1518	0.064*	
H14H	0.4663	−0.0677	−0.0822	0.064*	
H14I	0.6042	−0.071	−0.1166	0.064*	
C59	0.2641 (4)	0.05070 (17)	0.78344 (15)	0.0481 (9)	
H59A	0.3583	0.0615	0.7967	0.072*	
H59B	0.258	0.0423	0.7398	0.072*	
H59C	0.2352	0.0106	0.8047	0.072*	
C61	0.1786 (5)	0.1053 (2)	0.7965 (2)	0.0732 (13)	
Cu1	0.42728 (3)	0.267704 (15)	0.001112 (12)	0.01713 (9)	
N1	0.4453 (2)	0.24472 (10)	0.08931 (8)	0.0180 (5)	
N1A	0.3996 (2)	0.30174 (10)	−0.08428 (8)	0.0179 (5)	
N1B	0.6283 (2)	0.22133 (11)	−0.03035 (9)	0.0238 (5)	
N2	0.2294 (2)	0.30295 (10)	0.03186 (8)	0.0190 (5)	
N2A	0.5294 (2)	0.35941 (11)	0.01066 (8)	0.0198 (5)	
N2B	0.3779 (2)	0.16806 (11)	−0.02082 (8)	0.0215 (5)	
N3	0.1085 (5)	0.1501 (2)	0.8097 (2)	0.1129 (16)	
P1	0.93016 (7)	0.12973 (4)	0.10542 (3)	0.02482 (17)	
P2	0.03660 (8)	0.42966 (4)	0.84305 (3)	0.02755 (18)	
F1	0.78807 (15)	0.12757 (8)	0.06591 (7)	0.0332 (4)	
F2	0.98902 (18)	0.18154 (10)	0.05951 (7)	0.0497 (5)	
F3	0.98123 (19)	0.06887 (10)	0.06639 (9)	0.0575 (6)	
F4	0.8699 (2)	0.07824 (10)	0.15167 (7)	0.0652 (7)	
F5	0.87735 (18)	0.19075 (9)	0.14395 (8)	0.0497 (5)	
F6	1.07090 (19)	0.13123 (9)	0.14446 (8)	0.0535 (5)	
F7	0.18126 (16)	0.42809 (8)	0.88046 (7)	0.0390 (4)	
F8	0.02636 (18)	0.34994 (8)	0.85120 (7)	0.0404 (4)	
F9	0.10949 (18)	0.41899 (9)	0.78168 (6)	0.0397 (4)	
F10	0.0491 (2)	0.50900 (9)	0.83575 (7)	0.0510 (5)	
F11	−0.03529 (19)	0.44029 (10)	0.90485 (7)	0.0470 (5)	
F12	−0.10720 (18)	0.43035 (11)	0.80643 (7)	0.0530 (5)	

### Atomic displacement parameters (Å^2^)

**Table:**

	*U*^11^	*U*^22^	*U*^33^	*U*^12^	*U*^13^	*U*^23^
C1	0.0203 (14)	0.0184 (14)	0.0242 (13)	0.0031 (12)	−0.0003 (11)	−0.0012 (11)
C1A	0.0193 (14)	0.0213 (15)	0.0249 (13)	0.0001 (12)	−0.0006 (10)	−0.0044 (12)
C1B	0.0302 (17)	0.0305 (17)	0.0275 (14)	−0.0063 (14)	−0.0047 (12)	0.0021 (12)
C2	0.0265 (15)	0.0237 (15)	0.0226 (13)	0.0053 (13)	−0.0050 (11)	−0.0008 (12)
C2A	0.0258 (15)	0.0287 (16)	0.0183 (12)	0.0031 (13)	−0.0046 (11)	−0.0034 (12)
C2B	0.0232 (16)	0.052 (2)	0.0314 (15)	−0.0040 (15)	0.0008 (12)	0.0102 (15)
C3	0.0383 (17)	0.0225 (15)	0.0174 (12)	−0.0008 (13)	−0.0037 (12)	0.0009 (11)
C3A	0.0247 (15)	0.0303 (16)	0.0179 (12)	0.0037 (13)	0.0022 (11)	0.0017 (12)
C3B	0.0266 (17)	0.044 (2)	0.0242 (14)	0.0089 (15)	0.0045 (12)	0.0069 (14)
C4	0.0238 (15)	0.0133 (13)	0.0196 (12)	−0.0035 (11)	0.0033 (10)	−0.0020 (10)
C4A	0.0177 (14)	0.0220 (15)	0.0208 (12)	0.0052 (12)	0.0033 (10)	0.0000 (11)
C4B	0.0289 (16)	0.0288 (16)	0.0173 (12)	0.0088 (14)	0.0004 (11)	0.0032 (12)
C5	0.0319 (16)	0.0211 (15)	0.0209 (12)	−0.0051 (13)	0.0077 (11)	−0.0022 (12)
C5A	0.0223 (15)	0.0238 (15)	0.0293 (14)	0.0016 (13)	0.0066 (11)	0.0048 (12)
C5B	0.0417 (19)	0.0269 (17)	0.0245 (14)	0.0162 (15)	0.0030 (13)	−0.0030 (13)
C6	0.0283 (16)	0.0182 (14)	0.0267 (13)	−0.0025 (13)	0.0101 (11)	0.0000 (12)
C6A	0.0192 (14)	0.0185 (14)	0.0301 (14)	0.0027 (12)	0.0081 (11)	0.0010 (12)
C6B	0.0449 (19)	0.0181 (15)	0.0253 (14)	0.0088 (14)	0.0003 (13)	−0.0023 (12)
C7	0.0236 (15)	0.0138 (13)	0.0239 (13)	−0.0031 (12)	0.0053 (11)	−0.0033 (11)
C7A	0.0136 (14)	0.0181 (14)	0.0286 (13)	0.0038 (11)	0.0029 (10)	−0.0044 (11)
C7B	0.0355 (17)	0.0190 (14)	0.0171 (12)	0.0013 (13)	0.0000 (11)	−0.0005 (11)
C8	0.0204 (15)	0.0207 (15)	0.0368 (15)	−0.0003 (12)	0.0049 (12)	−0.0004 (13)
C8A	0.0203 (15)	0.0170 (14)	0.0367 (15)	0.0007 (12)	0.0018 (11)	−0.0070 (12)
C8B	0.049 (2)	0.0219 (16)	0.0271 (14)	−0.0041 (15)	−0.0003 (13)	−0.0005 (13)
C9	0.0199 (15)	0.0216 (15)	0.0385 (16)	0.0040 (12)	−0.0041 (12)	0.0022 (13)
C9A	0.0235 (15)	0.0270 (16)	0.0278 (14)	0.0007 (13)	−0.0040 (11)	−0.0116 (13)
C9B	0.0319 (17)	0.0358 (18)	0.0353 (15)	−0.0085 (15)	0.0079 (13)	0.0044 (14)
C10	0.0241 (15)	0.0206 (15)	0.0233 (13)	0.0000 (13)	−0.0045 (11)	0.0015 (12)
C10A	0.0270 (16)	0.0247 (15)	0.0193 (12)	0.0047 (13)	−0.0002 (11)	−0.0045 (12)
C10B	0.0306 (17)	0.0279 (16)	0.0258 (14)	0.0012 (14)	0.0075 (12)	0.0003 (12)
C11	0.0215 (14)	0.0106 (12)	0.0194 (12)	−0.0035 (11)	0.0013 (10)	−0.0029 (10)
C11A	0.0168 (14)	0.0167 (14)	0.0213 (12)	0.0035 (11)	0.0019 (10)	0.0001 (11)
C11B	0.0284 (16)	0.0182 (14)	0.0144 (11)	0.0056 (12)	0.0012 (11)	0.0024 (11)
C12	0.0208 (14)	0.0115 (12)	0.0168 (11)	−0.0034 (11)	0.0002 (10)	−0.0028 (10)
C12A	0.0147 (13)	0.0167 (14)	0.0199 (12)	0.0027 (11)	0.0019 (10)	−0.0008 (11)
C12B	0.0257 (15)	0.0193 (14)	0.0130 (11)	0.0067 (12)	0.0000 (10)	0.0013 (11)
C13	0.045 (2)	0.051 (2)	0.0232 (14)	0.0070 (17)	0.0122 (13)	0.0049 (14)
C13A	0.048 (2)	0.0389 (19)	0.0305 (15)	−0.0091 (16)	0.0043 (14)	0.0113 (14)
C13B	0.060 (2)	0.049 (2)	0.058 (2)	0.026 (2)	0.0203 (17)	−0.0133 (18)
C14	0.0410 (19)	0.0337 (18)	0.0382 (16)	0.0100 (16)	0.0205 (14)	0.0043 (14)
C14A	0.0313 (17)	0.0229 (16)	0.0429 (16)	−0.0037 (14)	0.0078 (13)	0.0012 (14)
C14B	0.063 (2)	0.0213 (17)	0.0431 (17)	0.0065 (16)	0.0057 (16)	−0.0087 (14)
C59	0.047 (2)	0.042 (2)	0.057 (2)	−0.0012 (18)	0.0131 (17)	0.0123 (17)
C61	0.072 (3)	0.049 (3)	0.098 (3)	−0.019 (3)	0.008 (3)	0.003 (3)
Cu1	0.02005 (17)	0.01618 (17)	0.01505 (15)	−0.00045 (14)	0.00014 (11)	−0.00118 (13)
N1	0.0195 (12)	0.0158 (11)	0.0186 (10)	0.0003 (9)	0.0006 (8)	−0.0018 (9)
N1A	0.0167 (11)	0.0161 (11)	0.0207 (10)	0.0006 (9)	0.0004 (8)	−0.0025 (9)
N1B	0.0301 (13)	0.0202 (13)	0.0204 (10)	−0.0002 (11)	−0.0029 (9)	−0.0003 (10)
N2	0.0210 (12)	0.0169 (12)	0.0189 (10)	0.0002 (10)	−0.0010 (9)	−0.0033 (9)
N2A	0.0181 (12)	0.0201 (12)	0.0213 (10)	0.0004 (10)	0.0016 (9)	−0.0033 (9)
N2B	0.0262 (13)	0.0224 (12)	0.0162 (10)	0.0039 (11)	0.0036 (9)	0.0002 (9)
N3	0.111 (4)	0.078 (3)	0.152 (4)	−0.009 (3)	0.023 (3)	−0.022 (3)
P1	0.0245 (4)	0.0236 (4)	0.0261 (3)	0.0015 (3)	−0.0006 (3)	0.0013 (3)
P2	0.0312 (4)	0.0258 (4)	0.0251 (4)	0.0061 (3)	−0.0020 (3)	−0.0028 (3)
F1	0.0238 (9)	0.0352 (10)	0.0400 (9)	0.0040 (8)	−0.0027 (7)	−0.0013 (8)
F2	0.0442 (11)	0.0628 (13)	0.0426 (9)	−0.0153 (10)	0.0059 (8)	0.0144 (9)
F3	0.0413 (11)	0.0521 (13)	0.0776 (13)	0.0195 (10)	−0.0091 (10)	−0.0309 (11)
F4	0.0966 (17)	0.0602 (14)	0.0367 (9)	−0.0424 (13)	−0.0132 (10)	0.0215 (10)
F5	0.0388 (11)	0.0458 (12)	0.0659 (11)	−0.0044 (9)	0.0132 (9)	−0.0276 (10)
F6	0.0442 (12)	0.0435 (12)	0.0688 (12)	0.0025 (9)	−0.0291 (10)	−0.0036 (10)
F7	0.0357 (10)	0.0354 (10)	0.0443 (9)	−0.0017 (8)	−0.0093 (8)	−0.0096 (8)
F8	0.0501 (12)	0.0291 (10)	0.0409 (9)	−0.0071 (9)	−0.0069 (8)	−0.0016 (8)
F9	0.0519 (11)	0.0383 (11)	0.0295 (8)	0.0069 (9)	0.0076 (8)	−0.0026 (8)
F10	0.0838 (15)	0.0231 (10)	0.0463 (10)	0.0131 (10)	0.0066 (9)	0.0018 (8)
F11	0.0539 (12)	0.0581 (13)	0.0299 (8)	0.0206 (10)	0.0096 (8)	0.0007 (8)
F12	0.0383 (11)	0.0735 (14)	0.0452 (10)	0.0160 (10)	−0.0131 (8)	−0.0013 (10)

### Geometric parameters (Å, º)

**Table:**

C1—N1	1.326 (3)	C10—H10	0.95
C1—C2	1.392 (3)	C10A—N2A	1.331 (3)
C1—H1	0.95	C10A—H10A	0.95
C1A—N1A	1.322 (3)	C10B—N2B	1.328 (4)
C1A—C2A	1.395 (3)	C10B—H10B	0.95
C1A—H1A	0.95	C11—N2	1.368 (3)
C1B—N1B	1.323 (3)	C11—C12	1.431 (3)
C1B—C2B	1.383 (4)	C11A—N2A	1.369 (3)
C1B—H1B	0.95	C11A—C12A	1.432 (3)
C2—C3	1.363 (4)	C11B—N2B	1.358 (3)
C2—H2	0.95	C11B—C12B	1.432 (4)
C2A—C3A	1.357 (4)	C12—N1	1.360 (3)
C2A—H2A	0.95	C12A—N1A	1.358 (3)
C2B—C3B	1.366 (4)	C12B—N1B	1.364 (3)
C2B—H2B	0.95	C13—H13A	0.98
C3—C4	1.409 (4)	C13—H13B	0.98
C3—H3	0.95	C13—H13C	0.98
C3A—C4A	1.414 (3)	C13A—H13D	0.98
C3A—H3A	0.95	C13A—H13E	0.98
C3B—C4B	1.408 (4)	C13A—H13F	0.98
C3B—H3B	0.95	C13B—H13G	0.98
C4—C12	1.403 (3)	C13B—H13H	0.98
C4—C5	1.451 (4)	C13B—H13I	0.98
C4A—C12A	1.405 (3)	C14—H14A	0.98
C4A—C5A	1.447 (4)	C14—H14B	0.98
C4B—C12B	1.404 (4)	C14—H14C	0.98
C4B—C5B	1.454 (4)	C14A—H14D	0.98
C5—C6	1.361 (4)	C14A—H14E	0.98
C5—C13	1.516 (3)	C14A—H14F	0.98
C5A—C6A	1.364 (4)	C14B—H14G	0.98
C5A—C13A	1.513 (3)	C14B—H14H	0.98
C5B—C6B	1.352 (4)	C14B—H14I	0.98
C5B—C13B	1.513 (4)	C59—C61	1.418 (6)
C6—C7	1.453 (3)	C59—H59A	0.98
C6—C14	1.509 (4)	C59—H59B	0.98
C6A—C7A	1.448 (3)	C59—H59C	0.98
C6A—C14A	1.505 (4)	C61—N3	1.176 (6)
C6B—C7B	1.454 (4)	Cu1—N1	2.0063 (19)
C6B—C14B	1.518 (4)	Cu1—N1A	2.0144 (19)
C7—C11	1.391 (3)	Cu1—N2A	2.091 (2)
C7—C8	1.411 (4)	Cu1—N2B	2.095 (2)
C7A—C11A	1.398 (4)	Cu1—N2	2.220 (2)
C7A—C8A	1.413 (3)	Cu1—N1B	2.333 (2)
C7B—C8B	1.405 (4)	P1—F2	1.5857 (18)
C7B—C11B	1.412 (4)	P1—F6	1.5889 (17)
C8—C9	1.363 (4)	P1—F3	1.5904 (19)
C8—H8	0.95	P1—F4	1.5923 (19)
C8A—C9A	1.367 (4)	P1—F5	1.5927 (18)
C8A—H8A	0.95	P1—F1	1.6053 (15)
C8B—C9B	1.367 (4)	P1—F1	1.6053 (15)
C8B—H8B	0.95	P2—F12	1.5888 (17)
C9—C10	1.392 (4)	P2—F9	1.5930 (17)
C9—H9	0.95	P2—F10	1.5954 (19)
C9A—C10A	1.391 (4)	P2—F11	1.5963 (17)
C9A—H9A	0.95	P2—F8	1.6030 (18)
C9B—C10B	1.395 (4)	P2—F7	1.6044 (16)
C9B—H9B	0.95	F1—F1	0.000 (6)
C10—N2	1.327 (3)		
			
N1—C1—C2	121.8 (2)	C5—C13—H13C	109.5
N1—C1—H1	119.1	H13A—C13—H13C	109.5
C2—C1—H1	119.1	H13B—C13—H13C	109.5
N1A—C1A—C2A	122.3 (3)	C5A—C13A—H13D	109.5
N1A—C1A—H1A	118.8	C5A—C13A—H13E	109.5
C2A—C1A—H1A	118.8	H13D—C13A—H13E	109.5
N1B—C1B—C2B	123.2 (3)	C5A—C13A—H13F	109.5
N1B—C1B—H1B	118.4	H13D—C13A—H13F	109.5
C2B—C1B—H1B	118.4	H13E—C13A—H13F	109.5
C3—C2—C1	119.5 (2)	C5B—C13B—H13G	109.5
C3—C2—H2	120.2	C5B—C13B—H13H	109.5
C1—C2—H2	120.2	H13G—C13B—H13H	109.5
C3A—C2A—C1A	119.6 (2)	C5B—C13B—H13I	109.5
C3A—C2A—H2A	120.2	H13G—C13B—H13I	109.5
C1A—C2A—H2A	120.2	H13H—C13B—H13I	109.5
C3B—C2B—C1B	119.4 (3)	C6—C14—H14A	109.5
C3B—C2B—H2B	120.3	C6—C14—H14B	109.5
C1B—C2B—H2B	120.3	H14A—C14—H14B	109.5
C2—C3—C4	120.4 (2)	C6—C14—H14C	109.5
C2—C3—H3	119.8	H14A—C14—H14C	109.5
C4—C3—H3	119.8	H14B—C14—H14C	109.5
C2A—C3A—C4A	120.2 (2)	C6A—C14A—H14D	109.5
C2A—C3A—H3A	119.9	C6A—C14A—H14E	109.5
C4A—C3A—H3A	119.9	H14D—C14A—H14E	109.5
C2B—C3B—C4B	119.7 (3)	C6A—C14A—H14F	109.5
C2B—C3B—H3B	120.1	H14D—C14A—H14F	109.5
C4B—C3B—H3B	120.1	H14E—C14A—H14F	109.5
C12—C4—C3	116.5 (2)	C6B—C14B—H14G	109.5
C12—C4—C5	119.7 (2)	C6B—C14B—H14H	109.5
C3—C4—C5	123.7 (2)	H14G—C14B—H14H	109.5
C12A—C4A—C3A	116.1 (2)	C6B—C14B—H14I	109.5
C12A—C4A—C5A	119.4 (2)	H14G—C14B—H14I	109.5
C3A—C4A—C5A	124.4 (2)	H14H—C14B—H14I	109.5
C12B—C4B—C3B	116.9 (3)	C61—C59—H59A	109.5
C12B—C4B—C5B	119.8 (3)	C61—C59—H59B	109.5
C3B—C4B—C5B	123.2 (3)	H59A—C59—H59B	109.5
C6—C5—C4	120.3 (2)	C61—C59—H59C	109.5
C6—C5—C13	123.9 (2)	H59A—C59—H59C	109.5
C4—C5—C13	115.8 (2)	H59B—C59—H59C	109.5
C6A—C5A—C4A	120.8 (2)	N3—C61—C59	177.5 (5)
C6A—C5A—C13A	121.5 (3)	N1—Cu1—N1A	172.89 (8)
C4A—C5A—C13A	117.7 (2)	N1—Cu1—N2A	95.07 (8)
C6B—C5B—C4B	120.6 (3)	N1A—Cu1—N2A	80.60 (8)
C6B—C5B—C13B	122.9 (3)	N1—Cu1—N2B	90.86 (8)
C4B—C5B—C13B	116.6 (3)	N1A—Cu1—N2B	94.88 (8)
C5—C6—C7	120.1 (2)	N2A—Cu1—N2B	162.88 (9)
C5—C6—C14	123.2 (2)	N1—Cu1—N2	78.35 (8)
C7—C6—C14	116.7 (2)	N1A—Cu1—N2	96.47 (8)
C5A—C6A—C7A	120.2 (2)	N2A—Cu1—N2	96.82 (8)
C5A—C6A—C14A	121.4 (2)	N2B—Cu1—N2	100.11 (8)
C7A—C6A—C14A	118.4 (2)	N1—Cu1—N1B	100.17 (8)
C5B—C6B—C7B	120.2 (3)	N1A—Cu1—N1B	85.38 (8)
C5B—C6B—C14B	122.0 (3)	N2A—Cu1—N1B	87.95 (8)
C7B—C6B—C14B	117.8 (3)	N2B—Cu1—N1B	75.18 (8)
C11—C7—C8	116.4 (2)	N2—Cu1—N1B	175.10 (8)
C11—C7—C6	120.0 (2)	C1—N1—C12	119.4 (2)
C8—C7—C6	123.6 (2)	C1—N1—Cu1	123.67 (17)
C11A—C7A—C8A	116.2 (2)	C12—N1—Cu1	116.96 (15)
C11A—C7A—C6A	119.5 (2)	C1A—N1A—C12A	118.6 (2)
C8A—C7A—C6A	124.3 (2)	C1A—N1A—Cu1	127.48 (18)
C8B—C7B—C11B	116.8 (3)	C12A—N1A—Cu1	113.71 (14)
C8B—C7B—C6B	123.4 (3)	C1B—N1B—C12B	118.1 (2)
C11B—C7B—C6B	119.8 (3)	C1B—N1B—Cu1	131.25 (19)
C9—C8—C7	120.1 (3)	C12B—N1B—Cu1	110.03 (17)
C9—C8—H8	120	C10—N2—C11	117.7 (2)
C7—C8—H8	120	C10—N2—Cu1	131.61 (17)
C9A—C8A—C7A	120.2 (3)	C11—N2—Cu1	110.37 (15)
C9A—C8A—H8A	119.9	C10A—N2A—C11A	118.0 (2)
C7A—C8A—H8A	119.9	C10A—N2A—Cu1	130.06 (18)
C9B—C8B—C7B	120.2 (3)	C11A—N2A—Cu1	111.44 (15)
C9B—C8B—H8B	119.9	C10B—N2B—C11B	119.0 (2)
C7B—C8B—H8B	119.9	C10B—N2B—Cu1	122.84 (19)
C8—C9—C10	119.5 (2)	C11B—N2B—Cu1	118.14 (18)
C8—C9—H9	120.2	F2—P1—F6	89.75 (10)
C10—C9—H9	120.2	F2—P1—F3	90.35 (11)
C8A—C9A—C10A	119.6 (2)	F6—P1—F3	90.90 (10)
C8A—C9A—H9A	120.2	F2—P1—F4	179.41 (13)
C10A—C9A—H9A	120.2	F6—P1—F4	90.54 (11)
C8B—C9B—C10B	119.4 (3)	F3—P1—F4	90.16 (12)
C8B—C9B—H9B	120.3	F2—P1—F5	89.56 (11)
C10B—C9B—H9B	120.3	F6—P1—F5	89.81 (10)
N2—C10—C9	122.6 (2)	F3—P1—F5	179.28 (11)
N2—C10—H10	118.7	F4—P1—F5	89.92 (11)
C9—C10—H10	118.7	F2—P1—F1	90.50 (9)
N2A—C10A—C9A	122.4 (2)	F6—P1—F1	179.54 (11)
N2A—C10A—H10A	118.8	F3—P1—F1	88.71 (9)
C9A—C10A—H10A	118.8	F4—P1—F1	89.22 (10)
N2B—C10B—C9B	122.2 (3)	F5—P1—F1	90.58 (9)
N2B—C10B—H10B	118.9	F2—P1—F1	90.50 (9)
C9B—C10B—H10B	118.9	F6—P1—F1	179.54 (11)
N2—C11—C7	123.7 (2)	F3—P1—F1	88.71 (9)
N2—C11—C12	116.3 (2)	F4—P1—F1	89.22 (10)
C7—C11—C12	119.9 (2)	F5—P1—F1	90.58 (9)
N2A—C11A—C7A	123.5 (2)	F1—P1—F1	0.00 (16)
N2A—C11A—C12A	116.1 (2)	F12—P2—F9	90.00 (9)
C7A—C11A—C12A	120.4 (2)	F12—P2—F10	90.66 (11)
N2B—C11B—C7B	122.3 (3)	F9—P2—F10	90.21 (10)
N2B—C11B—C12B	118.2 (2)	F12—P2—F11	90.46 (10)
C7B—C11B—C12B	119.5 (2)	F9—P2—F11	179.54 (11)
N1—C12—C4	122.4 (2)	F10—P2—F11	89.82 (10)
N1—C12—C11	117.9 (2)	F12—P2—F8	90.34 (10)
C4—C12—C11	119.7 (2)	F9—P2—F8	89.95 (10)
N1A—C12A—C4A	123.1 (2)	F10—P2—F8	178.99 (10)
N1A—C12A—C11A	117.3 (2)	F11—P2—F8	90.01 (10)
C4A—C12A—C11A	119.6 (2)	F12—P2—F7	179.26 (12)
N1B—C12B—C4B	122.6 (3)	F9—P2—F7	90.32 (9)
N1B—C12B—C11B	117.6 (2)	F10—P2—F7	90.00 (10)
C4B—C12B—C11B	119.7 (2)	F11—P2—F7	89.22 (9)
C5—C13—H13A	109.5	F8—P2—F7	89.00 (9)
C5—C13—H13B	109.5	F1—F1—P1	0 (10)
H13A—C13—H13B	109.5		
			
N1—C1—C2—C3	−1.1 (4)	C11—C12—N1—C1	179.4 (2)
N1A—C1A—C2A—C3A	−0.4 (4)	C4—C12—N1—Cu1	178.82 (18)
N1B—C1B—C2B—C3B	−0.1 (4)	C11—C12—N1—Cu1	−2.1 (3)
C1—C2—C3—C4	−0.2 (4)	N1A—Cu1—N1—C1	−137.6 (6)
C1A—C2A—C3A—C4A	−0.9 (4)	N2A—Cu1—N1—C1	−85.4 (2)
C1B—C2B—C3B—C4B	−0.2 (4)	N2B—Cu1—N1—C1	78.5 (2)
C2—C3—C4—C12	1.4 (4)	N2—Cu1—N1—C1	178.7 (2)
C2—C3—C4—C5	−177.1 (3)	N1B—Cu1—N1—C1	3.4 (2)
C2A—C3A—C4A—C12A	0.4 (4)	N1A—Cu1—N1—C12	44.0 (8)
C2A—C3A—C4A—C5A	−178.5 (3)	N2A—Cu1—N1—C12	96.21 (18)
C2B—C3B—C4B—C12B	0.3 (4)	N2B—Cu1—N1—C12	−99.86 (18)
C2B—C3B—C4B—C5B	−178.3 (2)	N2—Cu1—N1—C12	0.28 (17)
C12—C4—C5—C6	−2.6 (4)	N1B—Cu1—N1—C12	−174.96 (17)
C3—C4—C5—C6	175.9 (2)	C2A—C1A—N1A—C12A	2.3 (4)
C12—C4—C5—C13	178.0 (2)	C2A—C1A—N1A—Cu1	−171.71 (19)
C3—C4—C5—C13	−3.6 (4)	C4A—C12A—N1A—C1A	−2.9 (4)
C12A—C4A—C5A—C6A	−1.3 (4)	C11A—C12A—N1A—C1A	178.0 (2)
C3A—C4A—C5A—C6A	177.6 (3)	C4A—C12A—N1A—Cu1	171.92 (19)
C12A—C4A—C5A—C13A	177.0 (2)	C11A—C12A—N1A—Cu1	−7.2 (3)
C3A—C4A—C5A—C13A	−4.2 (4)	N1—Cu1—N1A—C1A	−124.7 (6)
C12B—C4B—C5B—C6B	−4.6 (4)	N2A—Cu1—N1A—C1A	−177.6 (2)
C3B—C4B—C5B—C6B	174.0 (2)	N2B—Cu1—N1A—C1A	19.1 (2)
C12B—C4B—C5B—C13B	175.0 (2)	N2—Cu1—N1A—C1A	−81.7 (2)
C3B—C4B—C5B—C13B	−6.4 (4)	N1B—Cu1—N1A—C1A	93.7 (2)
C4—C5—C6—C7	4.5 (4)	N1—Cu1—N1A—C12A	61.1 (7)
C13—C5—C6—C7	−176.1 (3)	N2A—Cu1—N1A—C12A	8.18 (17)
C4—C5—C6—C14	−175.3 (3)	N2B—Cu1—N1A—C12A	−155.17 (17)
C13—C5—C6—C14	4.1 (4)	N2—Cu1—N1A—C12A	104.05 (17)
C4A—C5A—C6A—C7A	2.6 (4)	N1B—Cu1—N1A—C12A	−80.50 (17)
C13A—C5A—C6A—C7A	−175.6 (2)	C2B—C1B—N1B—C12B	0.4 (4)
C4A—C5A—C6A—C14A	−177.6 (2)	C2B—C1B—N1B—Cu1	−169.13 (18)
C13A—C5A—C6A—C14A	4.2 (4)	C4B—C12B—N1B—C1B	−0.3 (3)
C4B—C5B—C6B—C7B	5.0 (4)	C11B—C12B—N1B—C1B	179.0 (2)
C13B—C5B—C6B—C7B	−174.6 (2)	C4B—C12B—N1B—Cu1	171.30 (17)
C4B—C5B—C6B—C14B	−175.4 (2)	C11B—C12B—N1B—Cu1	−9.3 (2)
C13B—C5B—C6B—C14B	5.1 (4)	N1—Cu1—N1B—C1B	−93.7 (2)
C5—C6—C7—C11	−1.9 (4)	N1A—Cu1—N1B—C1B	81.8 (2)
C14—C6—C7—C11	177.9 (2)	N2A—Cu1—N1B—C1B	1.1 (2)
C5—C6—C7—C8	178.4 (2)	N2B—Cu1—N1B—C1B	178.1 (2)
C14—C6—C7—C8	−1.8 (4)	N2—Cu1—N1B—C1B	−165.8 (7)
C5A—C6A—C7A—C11A	−2.2 (4)	N1—Cu1—N1B—C12B	96.13 (15)
C14A—C6A—C7A—C11A	177.9 (2)	N1A—Cu1—N1B—C12B	−88.35 (15)
C5A—C6A—C7A—C8A	177.3 (3)	N2A—Cu1—N1B—C12B	−169.08 (15)
C14A—C6A—C7A—C8A	−2.6 (4)	N2B—Cu1—N1B—C12B	7.95 (14)
C5B—C6B—C7B—C8B	177.8 (2)	N2—Cu1—N1B—C12B	24.1 (8)
C14B—C6B—C7B—C8B	−1.8 (4)	C9—C10—N2—C11	0.4 (4)
C5B—C6B—C7B—C11B	−0.2 (4)	C9—C10—N2—Cu1	−172.33 (19)
C14B—C6B—C7B—C11B	−179.8 (2)	C7—C11—N2—C10	0.7 (4)
C11—C7—C8—C9	−0.1 (4)	C12—C11—N2—C10	−177.2 (2)
C6—C7—C8—C9	179.7 (3)	C7—C11—N2—Cu1	174.89 (19)
C11A—C7A—C8A—C9A	−0.3 (4)	C12—C11—N2—Cu1	−3.0 (3)
C6A—C7A—C8A—C9A	−179.8 (2)	N1—Cu1—N2—C10	174.6 (2)
C11B—C7B—C8B—C9B	0.4 (4)	N1A—Cu1—N2—C10	−0.4 (2)
C6B—C7B—C8B—C9B	−177.7 (2)	N2A—Cu1—N2—C10	80.8 (2)
C7—C8—C9—C10	1.1 (4)	N2B—Cu1—N2—C10	−96.6 (2)
C7A—C8A—C9A—C10A	−0.4 (4)	N1B—Cu1—N2—C10	−112.4 (8)
C7B—C8B—C9B—C10B	1.6 (4)	N1—Cu1—N2—C11	1.53 (16)
C8—C9—C10—N2	−1.3 (4)	N1A—Cu1—N2—C11	−173.53 (16)
C8A—C9A—C10A—N2A	0.7 (4)	N2A—Cu1—N2—C11	−92.28 (17)
C8B—C9B—C10B—N2B	−1.9 (4)	N2B—Cu1—N2—C11	90.31 (16)
C8—C7—C11—N2	−0.9 (4)	N1B—Cu1—N2—C11	74.5 (8)
C6—C7—C11—N2	179.4 (2)	C9A—C10A—N2A—C11A	−0.3 (4)
C8—C7—C11—C12	177.0 (2)	C9A—C10A—N2A—Cu1	170.8 (2)
C6—C7—C11—C12	−2.8 (4)	C7A—C11A—N2A—C10A	−0.4 (4)
C8A—C7A—C11A—N2A	0.7 (4)	C12A—C11A—N2A—C10A	179.3 (2)
C6A—C7A—C11A—N2A	−179.7 (2)	C7A—C11A—N2A—Cu1	−173.1 (2)
C8A—C7A—C11A—C12A	−179.0 (2)	C12A—C11A—N2A—Cu1	6.6 (3)
C6A—C7A—C11A—C12A	0.6 (4)	N1—Cu1—N2A—C10A	6.1 (2)
C8B—C7B—C11B—N2B	−2.3 (3)	N1A—Cu1—N2A—C10A	−179.6 (2)
C6B—C7B—C11B—N2B	175.8 (2)	N2B—Cu1—N2A—C10A	−103.7 (3)
C8B—C7B—C11B—C12B	176.8 (2)	N2—Cu1—N2A—C10A	85.0 (2)
C6B—C7B—C11B—C12B	−5.1 (3)	N1B—Cu1—N2A—C10A	−93.9 (2)
C3—C4—C12—N1	−1.5 (4)	N1—Cu1—N2A—C11A	177.71 (17)
C5—C4—C12—N1	177.1 (2)	N1A—Cu1—N2A—C11A	−7.98 (16)
C3—C4—C12—C11	179.4 (2)	N2B—Cu1—N2A—C11A	67.9 (3)
C5—C4—C12—C11	−2.0 (4)	N2—Cu1—N2A—C11A	−103.45 (17)
N2—C11—C12—N1	3.5 (3)	N1B—Cu1—N2A—C11A	77.67 (17)
C7—C11—C12—N1	−174.5 (2)	C9B—C10B—N2B—C11B	0.1 (4)
N2—C11—C12—C4	−177.3 (2)	C9B—C10B—N2B—Cu1	−179.75 (19)
C7—C11—C12—C4	4.6 (4)	C7B—C11B—N2B—C10B	2.1 (3)
C3A—C4A—C12A—N1A	1.5 (4)	C12B—C11B—N2B—C10B	−177.0 (2)
C5A—C4A—C12A—N1A	−179.5 (2)	C7B—C11B—N2B—Cu1	−178.07 (17)
C3A—C4A—C12A—C11A	−179.4 (2)	C12B—C11B—N2B—Cu1	2.8 (3)
C5A—C4A—C12A—C11A	−0.4 (4)	N1—Cu1—N2B—C10B	73.80 (19)
N2A—C11A—C12A—N1A	0.2 (3)	N1A—Cu1—N2B—C10B	−102.00 (19)
C7A—C11A—C12A—N1A	179.9 (2)	N2A—Cu1—N2B—C10B	−175.8 (2)
N2A—C11A—C12A—C4A	−179.0 (2)	N2—Cu1—N2B—C10B	−4.52 (19)
C7A—C11A—C12A—C4A	0.7 (4)	N1B—Cu1—N2B—C10B	174.1 (2)
C3B—C4B—C12B—N1B	0.0 (3)	N1—Cu1—N2B—C11B	−106.03 (17)
C5B—C4B—C12B—N1B	178.7 (2)	N1A—Cu1—N2B—C11B	78.17 (17)
C3B—C4B—C12B—C11B	−179.4 (2)	N2A—Cu1—N2B—C11B	4.4 (4)
C5B—C4B—C12B—C11B	−0.7 (3)	N2—Cu1—N2B—C11B	175.65 (15)
N2B—C11B—C12B—N1B	5.2 (3)	N1B—Cu1—N2B—C11B	−5.73 (15)
C7B—C11B—C12B—N1B	−174.0 (2)	F2—P1—F1—F1	0.00 (4)
N2B—C11B—C12B—C4B	−175.4 (2)	F6—P1—F1—F1	0 (7)
C7B—C11B—C12B—C4B	5.4 (3)	F3—P1—F1—F1	0.00 (4)
C2—C1—N1—C12	1.0 (4)	F4—P1—F1—F1	0.00 (4)
C2—C1—N1—Cu1	−177.38 (19)	F5—P1—F1—F1	0.00 (4)
C4—C12—N1—C1	0.3 (4)	Cu1—N1—N2—N1A	−3.13 (5)

### Hydrogen-bond geometry (Å, º)

**Table:**

*D*—H···*A*	*D*—H	H···*A*	*D*···*A*	*D*—H···*A*
C1—H1···F1	0.95	2.3	3.149 (3)	148
C1*A*—H1*A*···N3^i^	0.95	2.74	3.489 (5)	136
C9—H9···F11^i^	0.95	2.62	3.270 (3)	126
C10—H10···F7^i^	0.95	2.54	3.358 (3)	145
C10—H10···F8^i^	0.95	2.59	3.471 (3)	155
C59—H59*C*···F4^ii^	0.98	2.3	3.265 (4)	170
C8*B*—H8*B*···F1^iii^	0.95	2.51	3.437 (3)	164
C3—H3···F8^iv^	0.95	2.47	3.392 (3)	163
C3*A*—H3*A*···F4^v^	0.95	2.62	3.557 (3)	169

Symmetry codes: (i) *x*, *y*, *z*−1; (ii) −*x*+1, −*y*, −*z*+1; (iii) −*x*+1, −*y*, −*z*; (iv) *x*+1/2, −*y*+1/2, *z*−1/2; (v) *x*−1/2, −*y*+1/2, *z*−1/2.
